# Effect of Government Guidelines and Corporate Governance on Telework Adoption and Occupational Health Measures in Taiwanese-Listed Companies

**DOI:** 10.1016/j.shaw.2024.04.004

**Published:** 2024-05-07

**Authors:** Chia-Jung Li, Louise E. Anthony, Tomohisa Nagata, Yawen Cheng, Ro-Ting Lin

**Affiliations:** 1 Department of Occupational Safety and Health, College of Public Health, China Medical University, Taichung, Taiwan; 2 International Master Program for Public Health, College of Public Health, China Medical University, Taichung, Taiwan; 3 Department of Occupational Health Practice and Management, Institute of Industrial Ecological Sciences, University of Occupational and Environmental Health, Kitakyushu, Japan; 4 Institute of Health Policy and Management, College of Public Health, National Taiwan University, Taipei, Taiwan

**Keywords:** COVID-19, Disclosure, Pandemic, Taiwan, Telework

## Abstract

**Background:**

Telework adoption in Taiwan has surged because of government guidelines during the COVID-19 pandemic. This study examined the disclosure practices of Taiwanese-listed companies, assessing their adherence to government telework guidelines and their correlation with corporate governance, focusing on occupational health measures.

**Methods:**

We conducted a guideline-adherent cohort analysis of the 2020 and 2021 sustainability reports of 295 Taiwanese-listed companies. We assessed their disclosure of corporate measures for teleworking in alignment with two government guidelines, specifically occupational health measures. Using the McNemar test and general estimating equation analysis, we compared the 2020 and 2021 responses and examined their associations with corporate governance rankings.

**Results:**

Telework adoption increased significantly from 2020 to 2021, with 68% of companies reporting new work modes. The mentioning of government guidelines also increased to 67% by 2021. Companies with higher governance rankings were more likely to adopt online occupational health measures, including occupational health services (RR = 2.03; 95% CI = 1.41–2.94; *p* < 0.001) and mental health promotion activities (RR = 2.01; 95% CI = 1.06–3.82; *p* = 0.032), than those with low rankings. Although on-site and online occupational health services increased, home workspace assessments did not.

**Conclusion:**

Our findings highlight significant upward trends in the disclosure of telework measures following the issuance of government guidelines. Corporate governance is significantly associated with the implementation of occupational health measures. Amid the evolution of teleworking, both government guidelines and corporate governance have become essential for shaping work arrangements and ensuring workforce well-being.

## 1 Introduction

Flexible work arrangements such as teleworking have gained significant attention in response to the global impact of the COVID-19 pandemic and beyond [1]. In the wake of the pandemic, the International Labor Organization (ILO) and governments worldwide endorsed teleworking as an effective strategy to protect employees’ health while ensuring business continuity [2,3]. Teleworking has been instrumental in mitigating the stress associated with commuting and concerns regarding COVID-19 transmission. In response to the global surge in teleworking precipitated by the COVID-19 pandemic, the interplay of responsibilities and rights among employers, workers, and governments has come under renewed scrutiny [4]. The Occupational Safety and Health Convention, 1981 (No. 155), and its Recommendation (No. 164) [5,6], alongside insights from the recent ILO reports [4], delineate a comprehensive framework emphasizing the collective duty to secure safe and healthful working conditions, which are universally applicable, including telework scenarios. These ILO standards mandate the member states to devise, execute, and periodically reassess a national occupational safety and health policy, ensuring its applicability to all work settings [[5], [6], [7]], telework included [4]. This framework is instrumental in adapting traditional workplace safety and health measures to the evolving nature of work, reflecting a global commitment to the well-being of the workforce in the digital age. This prompted many governments to establish guidelines and offer guidance to companies adjusting to this new operational mode [8,9]. Although Taiwan is not a member state, the Taiwanese government has vividly exemplified such a commitment through its strategic adaptation and implementation of comprehensive telework guidelines.

The Taiwanese government released two pivotal teleworking-related guidelines during the COVID-19 pandemic. The first guideline, known as the “Guidelines for Enterprise Planning of Business Continuity in Response to COVID-19” (hereafter referred to as Guideline 1), was announced by the Central Epidemic Command Center in March 2020; it encourages the adoption of telework or work from home arrangements to minimize infection risk [10]. The second guideline, titled “Guidelines on Occupational Safety and Health for Work from Home” (hereafter referred to as Guideline 2), was published by the Ministry of Labor in June 2021; it underscores the importance of hazard assessment, risk mitigation, and workers’ safety and health during telework [11]. Although the first guideline no longer applied after the threat of COVID-19 was downgraded, these two guidelines served as references for companies to align themselves with government and societal expectations during the pandemic and might still have lasting influences afterward.

The integration of telework practices can influence the environmental, social, and governance landscapes of a company. From a governance perspective, telework offers a strategic avenue for establishing robust protocols for data security and confidentiality [12]. Businesses can enhance their internal risk management by ensuring that employees receive appropriate training for safe and remote work. From a social perspective, embracing telework demonstrates a dedicated commitment toward safeguarding employee well-being while enhancing productivity [13,14]. Telework empowers employees to balance professional and personal commitments, leading to job satisfaction and work-life balance [15]. Adherence to government-mandated telework guidelines reflects a company’s alignment with broader community efforts to combat the pandemic, highlighting corporate governance in areas of compliance, business continuity, data privacy, and employee well-being [10,16]. Therefore, the implications of these guidelines extend beyond legal compliance and infection control to include these critical areas.

Corporate governance may influence the extent to which a company implements practices beyond its regulatory requirements. This encompasses setting strategic goals, ensuring organizational accountability, and fostering trust, transparency, and long-term investment [17], all of which underpin a company’s stable growth [18]. This extends to priorities, direction, and decisions related to occupational safety and health management [19,20]. Consequently, corporate governance can affect how a company discloses corporate goals, achievement processes, and performance indicators, shedding light on occupational health management [21].

Recently, companies typically disclose information that goes beyond the regulatory requirements in corporate social responsibility or sustainability reports (referred to as sustainability reports). The transparent disclosure of telework occupational safety and health practices in corporate reports helps stakeholders comprehend how companies safeguard their employees’ well-being amid an evolving pandemic-driven work landscape. Hence, sustainability reports have become important platforms for conveying information about corporate social responsibility.

In Taiwan, the Financial Supervisory Commission mandates that businesses actively participate in environmental and social initiatives to uphold sustainability reports. This requirement was reinforced after the 2008 financial crisis. Companies must now disclose their social responsibility performance if they engage in significant environmental and societal activities [22,23]. This requirement particularly applies to companies listed on the Taiwan Stock Exchange (TWSE) and Taipei Exchange (TPEx) [22,23]. To assist companies in meeting this requirement, TWSE and TPEx introduced the Sustainable Development Best Practice Principles for TWSE/TPEx Listed Companies [24], followed by the Rules Governing the Preparation and Filing of Corporate Social Responsibility Reports [25,26]. These rules stipulate that companies must prepare their reports in accordance with the Global reporting initiative (GRI) standards and sector disclosures issued by the GRI, as well as other relevant regulations based on their respective industry characteristics. This practice not only enhances transparency but also conveys a company’s dedication to its stakeholders [27,28].

Ensuring employee well-being, especially amid pandemic-induced changes in work arrangements, underscores the significance of transparent disclosure of telework-related occupational safety and health practices [11]. This disclosure maintains a safe teleworking environment to prevent discomfort and accidents and builds public trust by demonstrating the company’s commitment to employee well-being [29]. Although teleworking increased during the pandemic, the extent to which companies adhered to government guidelines remains uncertain. Understanding the adoption of telework, adherence to government guidelines, and the influence of corporate governance can provide valuable insights for companies operating in diverse international contexts. The experiences and practices observed in Taiwanese-listed companies can serve as a case study for understanding telework implementation in different regions and identifying best practices applicable on a global scale. Taiwan’s situation is especially pertinent due to its comprehensive and early response to telework challenges, including the prioritization of occupational safety and health. These practices suggest a model for developing effective telework policies and underscore the importance of a concerted effort between governments and corporations to enhance employee well-being. Thus, this study aimed to examine the disclosure practices of listed Taiwanese companies by assessing their compliance with government telework guidelines and their association with corporate governance. Particular focus was placed on the inclusion of occupational health measures.

## 2 Materials and methods

### 2.1 Study design and setting

We collected data on company measures over a two-year period following the announcement of the guidelines [10,11]. This guideline-adherent cohort study used sustainability reports obtained from the Market Observation Post System [30], which covers companies listed on the TWSE and the TPEx (hereafter referred to as listed companies). This study adhered to the Strengthening the Reporting of Observational Studies in Epidemiology guidelines for reporting observational studies.

### 2.2 Study period, companies, and study size

**Fig. 1:**
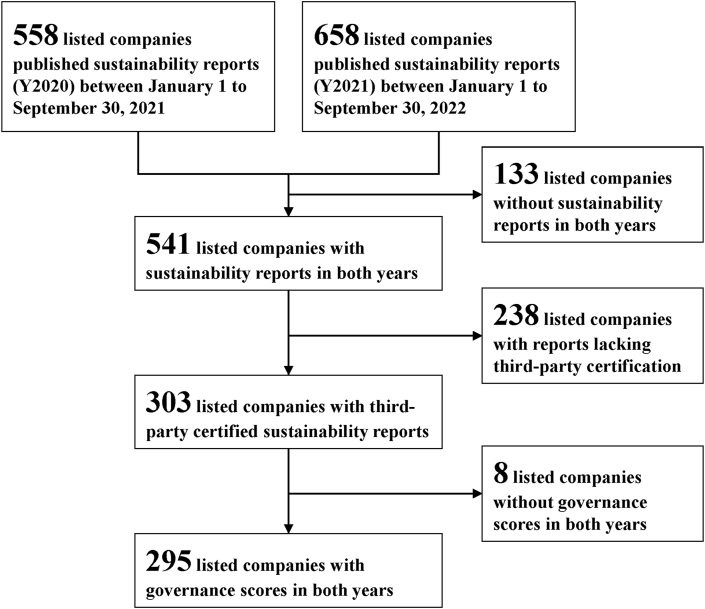
Data collection process and the number of companies in each stage.

Fig. 1 outlines the data collection process and the number of companies in each stage. We collected data from listed companies that published their activities report in 2020 from January 1 to September 30, 2021 (hereafter referred to as “Y2020 reports”) and their activity reports for 2021 in the period of January 1 to September 30, 2022 (hereafter referred to as “Y2021 reports”). The listed companies selected represent diverse industry sectors, including manufacturing, information, oil, gas, electricity, service, finance and insurance, building materials and construction, and shipping and transportation, and others. We included 558 listed companies that published Y2020 reports and 658 that published Y2021 reports. To enable year-on-year comparisons, we excluded 133 companies without sustainability reports in both years. To minimize observer bias, we filtered out sustainability reports lacking third-party certification, meaning that these reports had not been independently verified or assessed by external organizations for their reporting aligning with the GRI, which is a widely recognized framework for sustainability reporting. The exclusion resulted in 238 exclusions. We excluded eight companies without corporate governance scores in both years. Our analysis focuses on 295 companies with both Y2020 and Y2021 sustainability reports available for download from the Market Observation Post System [30].

### 2.3 Variables

This study comprised three key data components. The first part collected basic company characteristics such as company name, stock code, sustainability report publication date, industry category, and the number of employees from the Market Observation Post System. The second part comprised the corporate governance rankings of the listed companies. This study collected annual governance ranking data from the Corporate Governance Evaluation System published by the Corporate Governance Center [31]. The corporate governance of these companies was evaluated based on the following aspects: protecting shareholders’ rights and interests and treating them equitably, enhancing board composition and operations, increasing information transparency, promoting sustainable development, and extra credit and point-deduction indicators [31].

The third part focused on company teleworking activities extracted from sustainability reports. To assess compliance with Guidelines 1 and 2 and one governmental guideline regarding GRI 403 indicators [10,11,32], we formulated six questions. (The details are described in a previous publication [33].) Although no official English version of the guidelines exists, we have translated portions of the guidelines to ensure clarity (see Appendix 1).

We aimed to explore the working modes adopted during the COVID-19 pandemic in line with Guideline 1 recommendations to implement teleworking to reduce COVID-19 transmission and protect employees’ health [10]. This led to our first question (Q1): “Which working mode did the company adopt during the COVID-19 pandemic?” (referred to as “adopting new working modes”). To evaluate alignment with government guidelines [10], we posed our second question (Q2): “Does the sustainability report mention the company’s plans to implement flexible work hours, split operations, off-site work, or telework as per the guidelines?” (referred to as “mentioning government guidelines”).

For companies adopting telework and mentioning adherence to government guidelines, we expected their occupational safety and health actions to align with Guideline 2. This guideline provides a 27-item checklist for companies encompassing 6 aspects (necessary equipment and resources, home workspace, work-related facilities, physical and mental health management, education and training, and communication and management) [11]. Following a pilot survey and expert meetings, we structured four questions (Q3–Q6) to efficiently assess company disclosures [11,32]. Our study aimed to determine whether companies provide essential training to teleworkers to address occupational safety and health issues [11,32]. Formulated as our third question (Q3), we asked, “What kind of support is provided to teleworking employees?” (referred to as “providing telework support”), covering equipment, education, training, and work-related facilities. Recognizing the importance of communication in telework, we focused our fourth question (Q4) on communication channels to enhance efficiency and prevent extended working hours [11]. We asked, “Which online tools are used to enhance communication between supervisors and colleagues?” (referred to as “enhancing online communication”). Additionally, we investigated whether companies conduct risk assessments and identify hazards in home-based telework environments [11,32]. This led to the fifth question (Q5) on home workspace assessment, including temperature, lighting, noise, and home environment. We posed the question, “Has the company assessed employees’ home environments (considering noise, heat, radiation, lighting, etc.) following government workplace safety guidelines?” (referred to as “assessing home-based telework environment”). Finally, we assessed whether companies offer health promotion activities to reduce work-related stress for teleworkers [11,32]. The sixth question (Q6) asked, “What health promotion services are available to teleworking employees?” (referred to as “implementing occupational health measures”), covering physical and mental health management, mental health promotion, work-life balance, and online health promotion.

### 2.4 Data verification and keyword settings

To ensure the data reliability of the question items we constructed, we initially assessed the internal consistency reliability of all items in our questions, which aimed to measure the same concept. We conducted this assessment using sustainability reports of 28 semiconductor companies [33]. We selected the semiconductor industry because of its particular relevance in the context of Taiwan. In 2021, Taiwan had a total of 30 companies listed as components in the DJSI World, with 6 of them belonging to the semiconductor sector. Our analysis yielded a favorable Cronbach's α value of 0.811 [33], indicating that all items across the questions exhibited relatively high internal consistency. In addition, we employed Adobe Acrobat 9 Pro to conduct keyword searches of sustainability reports from 2020 to 2021. To evaluate the agreement between the human observers and the software, we calculated Cohen’s kappa coefficient. Table in Appendix 2 revealed that 95% of the individual surveyed indicators exhibited a moderate to almost perfect level of agreement [34].

### 2.5 Potential bias management

Cohort studies are susceptible to various biases, including reporting, recall, selection, and observer biases. To address these, we adopted several measures. We focused on sustainability reports certified by qualified accountants, known for their high quality and reduced report and recall biases. However, this approach introduced a potential selection bias because our sample did not cover all listed companies in Taiwan, limiting the generalizability of our findings. To minimize observer bias, we established a standardized review process, conducted a comprehensive training and pilot study [33], and used Adobe Acrobat for keyword searches as a secondary verification method. This ensured the objectivity of the analysis. To maintain data quality and consistency, we excluded 379 listed companies from our study owing to insufficient data: absence of sustainability reports (*n* = 133), lack of third-party certification (*n* = 238), or lack of governance scores (*n* = 8). Consequently, our final dataset included 295 Taiwanese-listed companies for statistical analysis.

### 2.6 Statistical analysis

To compare the responses across the 6 questions between 2020 and 2021, we performed the exact McNemar test. Additionally, we categorized companies into two groups based on their governance rankings: the top 5% and the remainder. To analyze the data for these questions and individual items, we conducted a General Estimating Equation analysis while adjusting for year, governance ranking, and number of employees. All the statistical analyses were performed using SAS version 9.4 (SAS Institute, Cary, NC, USA). Statistical significance was set at *p* < 0.05, employing a 2-sided test.

## 3 Results

Table 1 shows the number and percentage of companies implementing the measures for each question. Telework and split operations (Q1) showed a significant increase of 13% and 17%, respectively, from 2020 to 2021 (*p* < 0.001), with telework being the most prevalent at 48% in 2021. Alignment with government guidelines (Q2) showed a substantial increase (from 47% in 2020 to 67% in 2021, *p* < 0.001). Software and information security (15% in 2021) were the most common measures used to support telework (Q3), whereas other support measures remained below 10%. Online communication (Q4) primarily relied on videoconferencing software (32% in 2021), marking an 9% increase (*p* = 0.004). The assessment of home-based telework environments remained limited, as reported by only three companies (1%). The implementation of occupational health measures was high, particularly physical measures such as on-site occupational health services (58% in 2021), fitness facilities and activities (48% in 2021), and on-site mental health promotion activities (36% in 2021). Most of these occupational health measures exhibited significant increases, resulting in an overall increase of 21% (*p* < 0.001).

**Table 1:** Number and percentage of companies with teleworking measures in 2020 and 2021

Questions and items	2020		2021		*p*∗
**Adopting new working modes**					
Telework	102	(35%)	143	(48%)	<0.001
Split operation	58	(20%)	109	(37%)	<0.001
Work off-site	48	(16%)	49	(17%)	1.000
Flexible work time	20	(7%)	15	(5%)	0.359
Any of the above	148	(50%)	201	(68%)	<0.001
**Mentioning government guidelines**	140	(47%)	199	(67%)	<0.001
**Providing telework support**					
Software and information security	31	(11%)	43	(15%)	0.081
Computer equipment	13	(4%)	22	(7%)	0.108
Online video training	9	(3%)	22	(7%)	0.019
Telework manual	6	(2%)	15	(5%)	0.035
Online consultation	2	(1%)	4	(1%)	0.625
Any of the above	44	(15%)	75	(25%)	<0.001
**Enhancing online communication**					
Videoconferencing software	68	(23%)	93	(32%)	0.004
Line	10	(3%)	27	(9%)	<0.001
Voice dialing	12	(4%)	18	(6%)	0.238
E-mail	6	(2%)	16	(5%)	0.006
Any of the above	74	(25%)	119	(40%)	<0.001
**Assessing home-based telework environment**	0	(0%)	3	(1%)	—
**Implementing occupational health measures**					
On-site occupational health services	120	(41%)	170	(58%)	<0.001
Online occupational health services	29	(10%)	87	(29%)	<0.001
On-site mental health promotion activities	53	(18%)	106	(36%)	<0.001
Online mental health promotion activities	16	(5%)	27	(9%)	0.061
Fitness facilities and activities	99	(34%)	142	(48%)	<0.001
Training on taking rest breaks at work	0	(0%)	3	(1%)	—
Any of the above	134	(45%)	195	(66%)	<0.001

∗ Exact McNemar test.

After accounting for governance and number of employees, the significance of the items that showed increases in Table 1 also persisted in Table 2. The significant relative risk indicated that companies enhanced their efforts in these measures by a factor of 1.36 to 2.96 in 2021, compared with 2020.

**Table 2:** Associations between predictors and the outcome variables estimated using generalized estimating equations

Questions and items	Year (2021 *vs.* 2020)		Governance (high *vs.* low)		Number of employees (‰)	
	RR (95% CI)	*p*	RR (95% CI)	*p*	RR (95% CI)	*p*
**Adopting new working modes**						
Telework	1.39 (1.19–1.63)	<0.001	1.20 (0.98–1.49)	0.085	1.02 (1.01–1.02)	<0.001
Split operation	1.88 (1.47–2.40)	<0.001	1.38 (1.03–1.84)	0.032	1.00 (0.98–1.01)	0.672
Work off-site	1.01 (0.78–1.31)	0.925	1.18 (0.74–1.89)	0.482	1.02 (1.00–1.04)	0.044
Flexible work time	0.78 (0.48–1.27)	0.319	0.54 (0.23–1.24)	0.143	1.03 (1.00–1.06)	0.023
**Mentioning government guidelines**	1.42 (1.27–1.58)	<0.001	1.18 (1.02–1.36)	0.027	1.01 (1.01–1.02)	<0.001
**Providing telework support**						
Software and information security	1.37 (0.98–1.92)	0.069	1.27 (0.81–2.00)	0.299	1.03 (1.01–1.04)	0.002
Computer equipment	1.67 (0.94–2.98)	0.080	1.16 (0.58–2.32)	0.683	1.03 (1.01–1.05)	0.003
Online video training	2.40 (1.16–4.95)	0.019	2.12 (0.98–4.57)	0.055	1.02 (1.00–1.05)	0.054
Telework manual	2.46 (1.12–5.43)	0.026	1.91 (0.82–4.43)	0.134	1.02 (0.97–1.06)	0.458
Online consultation	1.56 (0.28–8.62)	0.612	4.34 (0.80–23.51)	0.088	1.06 (1.03–1.10)	<0.001
**Enhancing online communication**						
Videoconferencing software	1.36 (1.10–1.68)	0.004	1.15 (0.84–1.58)	0.386	1.02 (1.01–1.04)	0.002
Line	2.65 (1.54–4.57)	<0.001	1.65 (0.83–3.28)	0.151	1.00 (0.96–1.04)	0.874
Voice dialing	1.45 (0.81–2.57)	0.210	2.86 (1.47–5.58)	0.002	1.01 (0.99–1.04)	0.322
E-mail	2.59 (1.27–5.24)	0.009	2.03 (0.85–4.86)	0.113	1.00 (0.97–1.04)	0.813
**Assessing home-based telework environment**	—	—	1.81 (0.13–24.46)	0.655	1.01 (0.94–1.09)	0.754
**Implementing occupational health measures**						
On-site occupational health services	1.41 (1.24–1.61)	<0.001	1.28 (1.07–1.53)	0.006	1.01 (1.01–1.02)	<0.001
Online occupational health services	2.96 (2.15–4.06)	<0.001	2.03 (1.41–2.94)	<0.001	1.02 (1.01–1.04)	0.002
On-site mental health promotion activities	1.99 (1.58–2.51)	<0.001	1.15 (0.83–1.60)	0.403	1.02 (1.01–1.03)	<0.001
Online mental health promotion activities	1.63 (0.98–2.74)	0.062	2.01 (1.06–3.82)	0.032	1.03 (1.01–1.05)	<0.001
Fitness facilities and activities	1.42 (1.23–1.64)	<0.001	1.35 (1.11–1.65)	0.003	1.02 (1.01–1.02)	<0.001
Training on taking rest breaks at work	—	—	2.68 (0.24–29.78)	0.423	0.65 (0.41–1.02)	0.062

CI, confidence interval; RR, relative risk.

- The parameter was not included due to convergence issues in the model.

The implementation of occupational health measures increased, ranging from 1.41 to 2.96 (Table 2). The most substantial increase was observed in online occupational health services (RR = 2.96; 95% CI = 2.15–4.06; *p* < 0.001). Additionally, we found that companies with higher governance rankings were more likely to adopt occupational health measures, including online occupational services (RR = 2.03; 95% CI = 1.41–2.94; *p* < 0.001) and online mental health promotion activities (RR = 2.01; 95% CI = 1.06–3.82; *p* = 0.032). Overall, companies with more employees tended to implement more occupational health measures than smaller companies with fewer employees (RR = 1.01–1.03; *p* < 0.003).

## 4 Discussion

Recently, teleworking has gained popularity as an alternative to traditional in-person work arrangements where employees typically commute to a physical workplace [35]. Our study investigated the disclosure practices of 295 listed Taiwanese companies and assessed their alignment with government-issued telework guidelines in light of the evolving pandemic landscape. We also investigated the effect of corporate governance on these practices to support teleworking arrangements. Our findings revealed significant improvement in the responses to five of the six questions. These include adopting new work modes, mentioning government guidelines, providing telework support, enhancing online communication, and implementing occupational health measures. Notably, there were limited reports of companies conducting assessments of their employees’ home teleworking environments despite the recommendations outlined in Guideline 2. Our results also demonstrated that companies with higher governance rankings had a better likelihood to adopt online occupational health measures than those with smaller rankings.

Our analysis indicates a 18% increase in the number of companies adopting new work modes from 2020 to 2021. By 2021, 68% of the studied companies had adopted any one of the listed new work modes, with telework being the most prevalent (48%). The increased adoption of teleworking is an indicator of proactive corporate measures that align with the government Guideline 1, highlighting the preparedness of these companies. Their readiness is further bolstered by the presence of a dedicated national public health agency and a robust infrastructure, including integrated manual and digital solutions designed to facilitate the coordination of essential functions [36]. Therefore, this paradigm shift reflects companies’ diligent efforts to adapt to new working models and enhance workplace flexibility.

Regarding government guidelines, 67% of the companies surveyed mentioned Guideline 1 in their Y2021 reports, marking an increase of 20% from 47% in their Y2020 reports. Guideline 1 was specifically designed to mitigate infection rates and community transmission among workers [10]. These guidelines explicitly advised companies to implement teleworking to reduce the risk of inter-employee transmission. This highlights the influential role of government recommendations in shaping corporate strategies during the pandemic, suggesting the companies’ compliance with government guidelines [37].

When adopting the new work mode, employees faced the challenge of transitioning. To facilitate this shift, companies should initiate essential skills training programs [11]. Employees who received training adapted better than those who did not. Employees without training experienced initial challenges, such as stress, isolation, and longer working hours [38]. Government guidelines require companies to provide training on the information transmission equipment and software used for teleworking [11]. Our analysis revealed that videoconferencing software was the most widely used tool (32% in 2021). The combination of telework and video conferencing had benefits such as cost reduction and increased productivity, especially during disease outbreaks such as COVID-19, which spread through close contact [39]. Consequently, teleworking manuals and online training resources are increasingly provided to support teleworking adoption by our study companies. However, the utilization of these resources remained low in most categories (<10%), except for data security. This suggests the need for further enhancement and promotion of teleworking support tools.

Employers were advised to visit employees’ teleworking environments to ensure occupational safety and health as per Guideline 2 [11]. However, only a limited number of companies (1% in their Y2021 reports) mentioned visiting employees’ teleworking locations. This low rate could be attributed to employees’ reluctance to disclose personal matters [40], which poses significant challenges to employers. Additionally, logistical difficulties, such as the risk of COVID-19, hindered on-site visits. Notably, our study found that videoconferencing software is the most commonly used tool among the surveyed companies (32% in 2021). This software offers a practical alternative for assessing occupational safety and health by guiding employees in self-checks and providing remote consultations. Therefore, we recommend substituting on-site inspections with video conferencing, as it can reduce costs and save time while still enabling effective assessment and support for teleworking employees, in alignment with Guideline 2.

With the increasing adoption of telework, the need arises to provide occupational health measures. It has been reported that 47% of participants gained weight during telework [41], which is consistent with the findings from a 2020 survey [42]. While many participants changed their eating habits, reduced physical activity led to weight gain in approximately 28% of them [42]. The widespread adoption of telework during the COVID-19 pandemic has introduced health risks, including dietary changes, sleep disruptions, and potential addictions amplified by confinement and COVID-related anxiety [43]. Traditional on-site occupational health services in Taiwan posed challenges for remote workers. Government guidelines require the assessment of physical discomfort symptoms among teleworkers [11]. Our study observed significant increases in on-site (from 41% in 2020 to 58% in 2021) and online (from 10% in 2020 to 29% in 2021) occupational health services. Therefore, teleconsultation with occupational health professionals is recommended to mitigate risks and protect the well-being of teleworkers [44]. Our findings also revealed that companies with larger workforces tended to adopt a greater number of occupational health measures compared to smaller companies with fewer employees. This suggests that larger companies with a high number of employees who are not physically present have transitioned their occupational health activities to an online mode.

Mental health concerns among teleworkers have become increasingly significant, particularly in the early stages of the pandemic [45]. Various factors contribute to mental health challenges faced by teleworkers. Poor home working environments, characterized by issues such as inadequate ventilation and difficulties in staying hydrated and resting, have been linked to employee mental health issues [46]. Additionally, teleworkers often grapple with conflicts between their personal and work lives owing to blurred boundaries when working and living in the same space [47,48]. Isolation is a commonly cited challenge that deters individuals from fully embracing teleworking from home [49], emphasizing the importance of maintaining connections with colleagues for productivity and job satisfaction [50]. Organizations have recognized the need for ongoing mental health support initiatives to address these concerns. These initiatives include transitioning from on-site to online counseling services, providing guidance on mental health information, and establishing online support networks involving supervisors and colleagues [51,52]. We observed increases in both on-site (from 18% in 2020 to 36% in 2021) and online (from 5% in 2020 to 9% in 2021) mental health promotion initiatives. These findings emphasize the measures taken to reduce work-related stress and enhance teleworkers’ well-being within the surveyed companies, even amid the challenges posed by the pandemic.

In the realm of environmental, social, and governance considerations, corporate governance is a fundamental pillar, intimately connected to corporate performance. A previous study has shown a significant positive correlation between better governance practices and improved operating performance [53]. Aligning with the previous research, our study also found that strong corporate governance, reflected in higher governance rankings, was associated with greater adoption of online occupational health measures, including online occupational health services (RR = 2.03) and mental health promotion activities (RR = 2.01) during the evolution of teleworking. Corporate governance is pivotal in shaping a company’s approach to telework, emphasizing strategic decision-making, business continuity, employee well-being, and societal compliance [17,54]. Research also indicates that companies with engaged boards and management exhibit better occupational safety and health performance [55,56]. Thus, if occupational safety and health are material concerns, corporate governance can significantly affect strategy and performance [57]. Firms with strong governance consider occupational safety and health risks in their decision-making [58]. It fosters transparency, accountability, and commitment to employee health and extends to decisions regarding occupational health practices [18]. Our findings underscore the significant influence of corporate governance on the ethos of telework adoption and dedication to employee health, enabling better navigation of telework complexities and informed decisions for employee well-being and safety as telework continues to evolve in response to global challenges.

In the aftermath of the COVID-19 pandemic, the global adoption of telework has necessitated a thorough reevaluation of occupational health practices. The Eurofound study, encompassing 15 countries across Europe, North America, and Asia, reflects this shift, delineating telework’s advantages, such as enhanced work-life balance and productivity, against its challenges, notably increased work intensity and the merging of work and personal life [59]. This research underlines the critical role of government-led initiatives and collective agreements in establishing effective telework frameworks [59]. In line with these findings, our study signals a growing trend toward flexible working arrangements and highlights the essential collaborative efforts of governments and corporations in adapting to this change. A related investigation into the teleworking experiences of Spanish civil servants during the pandemic pinpoints key elements for successful telework adoption, including equipment investment, information and communications technology (ICT) training, and work-life balance policies [60], consistent with the guidelines of Taiwanese Guideline 2 [11]. Our analysis in Taiwan accentuates the pressing necessity for focused occupational health management in telecommuting, underlining its vital importance for the evolution of workplace practices, and the development of corporate policy, necessitating strong governance. Furthermore, the Organisation for Economic Co-operation and Development report anticipates telework as a lasting component of the post-pandemic work environment, emphasizing the crucial role of public policies and social partnerships in fostering productive telework practices [61]. Our findings validate these insights and stress the need for concerted government action and sound corporate governance to provide comprehensive telework support at the corporate, national, and international levels, thus affirming its significance for global research and policy development.

This study had a few limitations. First, it focused exclusively on listed companies. Consequently, our findings may not fully represent companies falling outside our inclusion criteria or smaller and medium-sized enterprises not listed on public stock exchanges. However, it is important to acknowledge that the included companies likely possess greater resources, making them better equipped to implement flexible work arrangements, provide telework support, and establish occupational health measures than those excluded. Thus, the observed percentage of non-participating companies may be lower. Second, our primary data source relied on sustainability reports, which introduces potential limitations. While sustainability reports offer valuable insights into companies’ sustainability and environmental, social, and governance practices, they are self-reported documents and are thus susceptible to reporting bias. This bias arises from the possibility that companies may choose to emphasize positive practices while under-reporting or omitting negative ones. To address this concern, we applied rigorous criteria in our report selection process, including requirements for consecutive-year reports, third-party verification, and timely publication. These criteria were used to enhance the reliability and validity of the data used in our analysis. A potential avenue for future research could involve exploring the disparities between mandatory reporting by listed companies and voluntary reporting by other companies.

Despite the limitations discussed, our study, rooted in the Taiwanese context, offers insights with potential global relevance, particularly in the context of the increasing popularity of telework. It highlights the need for comprehensive national policies on occupational safety and health, informed by the principles of the ILO conventions and recommendations [[5], [6], [7]], as demonstrated by Taiwan’s swift actions to formulate telework guidelines and emphasize occupational health. These measures reflect a high level of preparedness and innovative policymaking that could serve as valuable guidance for other countries navigating the intricacies of telework. Our findings indicate that creating safe and healthy work environments in the era of telework requires good corporate governance. Although our study did not detect a direct interaction effect between corporate governance and government policy over time, Taiwan’s proactive steps in formulating telework guidelines and emphasizing occupational safety and health provide a constructive path for managing telework challenges [10,11]. Taiwan’s experience, illustrating the important roles of both government and corporations in fostering employee well-being, provides practical insights and strategies that other nations and companies can adapt to, and contributing to the broader conversation on shaping resilient and supportive work environments in an evolving telework landscape.

The increased telework during the pandemic led us to investigate its impact on the disclosure of occupational health measures in Taiwan’s listed companies. Our findings indicate a significant increase in information disclosure, especially in online occupational health services and mental health promotion activities, emphasizing the influence of both government guidance and corporate governance. This reflects the adaptability of the surveyed companies in implementing flexible occupational health services, collectively contributing to teleworking and employees’ well-being and safety in line with labor health protection regulations. Amid the evolution of teleworking, our study highlights the important role played by government guidelines and corporate governance in driving the adoption of online occupational health services and mental health promotion activities, thereby enhancing employee well-being.

## Funding

This work was supported by the National Science and Technology Council, Taiwan (grant number NSTC 111-2314-B-039-020-MY2) and 10.13039/501100012544China Medical University, Taiwan (grant numbers CMU112-S-26 and CMU112-MF-76). The funding source had no role in the study design, data collection, data analysis, data interpretation, writing of the manuscript, or decision to submit the paper for publication.

## CRediT authorship contribution statement

**Chia-Jung Li:** Conceptualization, Data curation, Formal analysis, Writing – original draft. **Louise E. Anthony:** Writing – review & editing, Writing – original draft. **Tomohisa Nagata:** Writing – review & editing. **Yawen Cheng:** Writing – review & editing. **Ro-Ting Lin:** Conceptualization, Data curation, Formal analysis, Funding acquisition, Methodology, Project administration, Supervision, Visualization, Writing – original draft, Writing – review & editing.

## Conflicts of interest

The authors declare that they have no conflict of interest.

## References

[1] Silver H. (2023). Working from home: before and after the pandemic. Contexts.

[2] International Labour Organization (2020).

[3] Allain-Dupré D., Chatry I., Kornprobst A., Michalun M.-V. (2020).

[4] World Health Organization, International Labour Organization (2021).

[5] (1981). C155 - occupational safety and health convention.

[6] (1981). R164 - occupational safety and health recommendation.

[7] (2006). C187 - promotional framework for occupational safety and health convention.

[8] Lockton Global Compliance News (2023). New remote working legislation around the world [Updated]. https://globalnews.lockton.com/new-remote-working-legislation-around-the-world/.

[9] International Labour Organization (2021).

[10] Ministry of Health and Welfare, Taiwan (2020). Guidelines for enterprise planning of business continuity in response to COVID-19 Taiwan. https://www.cdc.gov.tw/File/Get/l53t2OP2wfHGqwEjISd4vw.

[11] Occupational Safety and Health Administration, Ministry of Labor, Taiwan (2021). Guidelines on occupational safety and health for work from home. https://laws.mol.gov.tw/FLAW/FLAWDAT0202.aspx?id=FL096766.

[12] International Labour Organization (2020).

[13] Hamar B., Coberley C., Pope J.E., Rula E.Y. (2015). Well-being improvement in a midsize employer changes in well-being, productivity, health risk, and perceived employer support after implementation of a well-being improvement strategy. J Occup Environ Med.

[14] Parker S.K., Grote G. (2022). Automation, algorithms, and beyond: why work design matters more than ever in a digital world. Appl Psychol.

[15] Thulin E., Vilhelmson B., Johansson M. (2019). New telework, time pressure, and time use control in everyday life. Sustainability.

[16] De-la-Calle-Durán M.C., Rodríguez-Sánchez J.L. (2021). Employee engagement and wellbeing in times of COVID-19: a proposal of the 5Cs model. Int J Environ Res Public Health.

[17] EU-OSHA. Governance of EU-OSHA [Available from: https://osha.europa.eu/en/about-eu-osha/governance-eu-osha.

[18] Organisation for Economic Cooperation and Development (OECD) (2015).

[19] Lornudd C., Tafvelin S., von Thiele Schwarz U., Bergman D. (2015). The mediating role of demand and control in the relationship between leadership behaviour and employee distress: a cross-sectional study. Int J Nurs Stud.

[20] Lornudd C., Frykman M., Stenfors T., Ebbevi D., Hasson H., Sundberg C.J., von Thiele Schwarz U. (2021). A champagne tower of influence: an interview study of how corporate boards enact occupational health and safety. Saf Sci.

[21] ASX Corporate Governance Council (2007).

[22] Financial Supervisory Commission of Taiwan. Legislative history of Regulations Governing Information to be Published in Public Offering and Issuance Prospectuses [2023 Sep 30]. Available from: https://law.moj.gov.tw/ENG/LawClass/LawHistory.aspx?pcode=G0400019.

[23] Financial Supervisory Commission of Taiwan (2008 Dec 25). Regulations Governing Information to Be Published in Public Offering and Issuance Prospectuses. https://law.moj.gov.tw/LawClass/LawOldVer.aspx?pcode=G0400019&lnndate=20081225&lser=001.

[24] Taiwan Stock Exchange (TWSE) and Taipei Exchange (TPEx). Legislative/regulatory history of Sustainable Development Best Practice Principles for TWSE/TPEx Listed Companies [2023 Oct 5]. Available from: https://eng.selaw.com.tw/LawHistory.aspx?LawID=FL052368&ModifyDate=1090213.

[25] Taiwan Stock Exchange (TWSE) (2014 Nov 26). Rules Governing the Preparation and Filing of Corporate Social Responsibility Reports by TWSE Listed Companies. http://www.selaw.com.tw/LawArticle.aspx?LawID=G0100517&ModifyDate=1031126.

[26] Taipei Exchange (TPEx) (2014 Dec 4). Rules Governing the Preparation and Filing of Corporate Social Responsibility Reports by TPEx Listed Companies. http://www.selaw.com.tw/LawContent.aspx?LawID=G0101624&ModifyDate=1031204.

[27] Burton W.N., Conti D.J., Chen C.Y., Schultz A.B., Edington D.W. (1999). The role of health risk factors and disease on worker productivity. J Occup Environ Med.

[28] Fernandez-Feijoo B., Romero S., Ruiz S. (2014). Effect of stakeholders’ pressure on transparency of sustainability reports within the GRI framework. J Bus Ethics.

[29] Nagata T., Nakata A., Mori K., Maruyama T., Kawashita F., Nagata M. (2017). Occupational safety and health aspects of corporate social responsibility reporting in Japan from 2004 to 2012. BMC Public Health.

[30] Taiwan Stock Exchange (TWSE), Taipei Exchange (TPEx). Market Observation Post System [2023 Sep 30]. Available from: https://mops.twse.com.tw/mops/web/t100sb11.

[31] Corporate Governance Center. Corporate Governance Evaluation System [Available from: https://cgc.twse.com.tw/evaluationCorp/listCh.

[32] Occupational Safety and Health Administration, Ministry of Labor, Taiwan (2022). Guidance on OSH indicators for CSR eporting towards SDGs. https://www.osha.gov.tw/48110/48207/134603/134615/134674/.

[33] Li C.J. (2023).

[34] McHugh M.L. (2012). Interrater reliability: the kappa statistic. Biochem Med.

[35] Allen T.D., Golden T.D., Shockley K.M. (2015). How effective is telecommuting? Assessing the status of our scientific findings. Psychol Sci Public Interest.

[36] Summers J., Cheng H.Y., Lin H.H., Barnard L.T., Kvalsvig A., Wilson N., Baker M.G. (2020). Potential lessons from the Taiwan and New Zealand health responses to the COVID-19 pandemic. Lancet Reg Health West Pac.

[37] Wirba A.V. (2023).

[38] Montreuil S., Lippel K. (2003). Telework and occupational health: a Quebec empirical study and regulatory implications. Saf Sci.

[39] Okereafor K., Manny P. (2020). Understanding cybersecurity challenges of telecommuting and video conferencing applications in the COVID-19 pandemic. Int J IT Eng.

[40] Katsabian T. (2020 Sep 1). The telework virus: how the COVID-19 pandemic has affected telework and exposed its implications for privacy and equality. SSRN.

[41] Guler M.A., Guler K., Gulec M.G., Ozdoglar E. (2021). Working from home during a pandemic: investigation of the impact of COVID-19 on employee health and productivity. J Occup Environ Med.

[42] Flanagan E.W., Beyl R.A., Fearnbach S.N., Altazan A.D., Martin C.K., Redman L.M. (2021). The impact of COVID-19 stay-at-home orders on health behaviors in adults. Obesity.

[43] Tavares A.I. (2017). Telework and health effects review. Int J Healthc.

[44] Bouziri H., Smith D.R.M., Descatha A., Dab W., Jean K. (2020). Working from home in the time of COVID-19: how to best preserve occupational health?. J Occup Environ Med.

[45] Hall C.E., Davidson L., Brooks S.K., Greenberg N., Weston D. (2023). The relationship between homeworking during COVID-19 and both, mental health, and productivity: a systematic review. BMC Psychol.

[46] Sasaki N., Kuroda R., Mikami Y., Tsuno K., Imamura K., Nishi D., Kawakami N. (2023). Working environment at home and mental health in employees working from home in Japan during COVID-19 pandemic: a cross-sectional study. J Occup Health.

[47] Delanoeije J., Verbruggen M., Germeys L. (2019). Boundary role transitions: a day-to-day approach to explain the effects of home-based telework on work-to-home conflict and home-to-work conflict. Hum Relat.

[48] Greer T.W., Payne S.C. (2014). Overcoming telework challenges: outcomes of successful telework strategies. Psychol Manag J.

[49] Bloom N., Liang J., Roberts J., Ying Z.J. (2015). Does working from home work? Evidence from a Chinese experiment. Q J Econ.

[50] Golden T.D., Gajendran R.S. (2019). Unpacking the role of a telecommuter’s job in their performance: examining job complexity, problem solving, interdependence, and social support. J Bus Psychol.

[51] Oakman J., Kinsman N., Stuckey R., Graham M., Weale V. (2020). A rapid review of mental and physical health effects of working at home: how do we optimise health?. BMC Public Health.

[52] Knardahl S., Christensen J.O. (2022). Working at home and expectations of being available: effects on perceived work environment, turnover intentions, and health. Scand J Work Environ Health.

[53] Bhagat S., Bolton B. (2008). Corporate governance and firm performance. J Corp Fin.

[54] Pavarin A.A., Sigahi T.F.A.C., Moraes GHSMd, Leal Filho W., Rampasso I.S., Anholon R. (2022). Analysis of corporate governance, organisational resilience and sustainable practices developed by Brazilian companies during the COVID-19 pandemic: an exploratory study. World.

[55] Bunn I.I.I.W.B., Pikelny D.B., Slavin T.J., Paralkar S. (2001). Health, safety, and productivity in a manufacturing environment. J Occup Environ Med.

[56] Joss N., Dupré-Husser E., Cooklin A., Oldenburg B. (2017). The emergence of integrated approaches to worker health, safety and wellbeing in Australia. Aust J Prim Health.

[57] Smallman C., John G. (2001). British directors perspectives on the impact of health and safety on corporate performance. Saf Sci.

[58] Lo D. (2012). OHS stewardship – integration of OHS in corporate governance. Procedia Eng.

[59] Messenger J., Llave O.V., Gschwind L., Boehmer S., Vermeylen G., Wilkens M. (2017).

[60] Ortiz-Lozano J.M., Martínez-Morán P.C., de Nicolás V.L. (2022). Teleworking in the public administration: an analysis based on Spanish civil servants’ perspectives during the pandemic. Sage Open.

[61] Organisation for Economic Co-operation and Development (OECD) (2020).

