# Supplementary material for: Effect of Government Guidelines and Corporate Governance on Telework Adoption and Occupational Health Measures in Taiwanese-Listed Companies
Source: Saf Health Work. 2024 May 7;15(2):164–71. doi: 10.1016/j.shaw.2024.04.004 (PMC11255928; doi:10.1016/j.shaw.2024.04.004)
Supplement: Multimedia component 1 [file mmc1.docx]

# **Appendix 1**

**Table of content**

[**Guideline 1 2**](#_Toc162277194)

[**Guideline 2 7**](#_Toc162277195)

# **Guideline 1**

**Guidelines for Enterprise Planning of Business Continuity in Response to COVID-19**

**Version: 2020.03.05**

1. **Epidemic situation**

The novel coronavirus (SARS-CoV-2) is the pathogen responsible for severe acute respiratory syndrome coronavirus 2 (COVID-19), commonly known as Wuhan pneumonia. Most human coronaviruses primarily spread through direct contact with virus-containing secretions or droplets. Infected individuals commonly experience respiratory symptoms such as nasal congestion, runny nose, cough, fever, and other typical upper respiratory tract infection symptoms. Additionally, a minority may develop more severe respiratory diseases, including pneumonia, which can be fatal.

The pneumonia epidemic triggered by SARS-CoV-2 originated in the Wuhan region of mainland China at the end of 2019. It has since proliferated throughout China and numerous countries worldwide, with confirmed cases comprising both imported and locally transmitted cases in China. For the latest epidemic updates, please visit the website of the Taiwan Centers for Disease Control (CDC).

The current domestic epidemic situation is characterized by sporadic community infections. However, if the risk of the epidemic escalates in the future and community transmission ensues, the challenges and impacts faced by enterprises will vary in severity. Therefore, this guideline is formulated to provide enterprises with risk assessment and response strategies tailored to both “sporadic community infection” and “community transmission.” This approach enables enterprises to sustain their business operations and mitigate losses to the greatest extent possible.

1. **Risk and impact assessment**

As COVID-19 is a newly emerging infectious disease, ongoing research into its transmissibility, severity, and other characteristics makes it challenging to accurately gauge the extent of infection, severity of cases, and mortality rates. At present, the domestic epidemic has not seen community transmission. Confirmed cases primarily originate from overseas travel, family gatherings, contact histories, or sporadic cases within healthcare settings or the community. The epidemic remains within manageable bounds. Nevertheless, the operations of certain manufacturing or service industries have been progressively impacted due to the evolving epidemic situation in mainland China and other countries.

- 1. **Current stage (sporadic community infections)**

Risks and potential impacts on corporate operations:

- **Personnel attendance**: Potential staff shortages may arise due to employees with travel or contact history experiencing fever or respiratory symptoms, employees being quarantined, or their family members being quarantined. Additionally, colleagues may require quarantine, further impacting staffing levels.
- **Business expansion**: Restrictions on travel, including travel warnings and reduced or suspended flights or transportation services, may hinder business expansion efforts. This could result in delays in meeting time schedules, delayed shipments, potential fines from customers due to late deliveries, or the need to seek alternative suppliers.
- **Production operations**: Company operations may face disruptions due to reduced or suspended transportation services, leading to shortages of raw materials and delayed deliveries. Furthermore, employee attendance issues may affect production schedules, potentially resulting in financial scheduling difficulties. The impact of these disruptions could persist for 2-3 months.
  1. **The epidemic enters the stage of sustained or widespread community transmission**

Risks and potential impacts on corporate operations:

- **Personnel attendance:** The presence of suspected cases within the company, employees unable to work due to confirmed cases, and other personnel required to quarantine or manage their health at home may lead to a severe shortage of company personnel. Possible cluster infections could result in the necessity to close offices, workplaces, or operational premises, rendering them unable to operate normally.
- **Business expansion:** Company operations may be interrupted due to the suspension or reduction of flights, shipping, or transportation services. Travel warnings may prevent participation in exhibitions or travel activities, leading to a temporary cessation of business activities. Reduced production lines or shutdowns could result in fines or trigger long-term order cancellations. Additionally, the inability to repay loans may result in bank interest arrears.
- **Production operations:** Insufficient inventory materials, shortages of raw materials and parts, and delays in shipments or deliveries from upstream and downstream suppliers may cause production halts. The stability of basic infrastructure such as water, electricity, oil, and air conditioning for the company’s operations may also be compromised. Logistics disruptions could affect shipments or lead to capital accumulation issues, impacting the company’s finances and resulting in financial turnover issues. The potential impact of these disruptions could last 2-3 months or even half a year.

1. **Countermeasures**

Enterprises should assign specific personnel responsible for epidemic prevention and establish an epidemic response unit. This unit will be tasked with monitoring epidemic developments, promoting awareness, preparing epidemic supplies, managing hygiene, monitoring personnel health, reporting epidemic updates, and preparing response strategies. The scope of epidemic prevention measures should encompass enterprise employees (including foreign workers), contractors, customers, and personnel stationed abroad.

- 1. **Recommended response strategies for enterprises at the current stage (sporadic community infection)**
     1. **Anti-epidemic suggestions**

1. **Encourage employees with fever or acute respiratory symptoms to stay home proactively**

- Recommend that employees with acute respiratory symptoms refrain from returning to work until their symptoms, including fever, have improved for at least 24 hours without the use of fever-reducing or other symptom-relieving medications (such as cough medicine).
- Adjust leave policies to waive the requirement for a doctor’s diagnosis for employees suffering from acute respiratory diseases. Medical institutions may be overwhelmed, making it challenging to obtain timely certificates. Additionally, to minimize the risk of infection, employees with mild illnesses should avoid unnecessary hospital visits.
- Maintain flexible leave policies to accommodate employees needing to care for sick family members. Employers should understand that an increased number of employees may need to stay home to attend to sick children or relatives.
- Ensure that the company’s leave policies are flexible and compliant with government regulations, and communicate these regulations clearly to employees.

1. **Personal and workplace hygiene management for employees with fever or acute respiratory symptoms**

- If employees develop fever or acute respiratory symptoms (such as cough or shortness of breath) while at work, they should promptly inform their supervisor. The company should instruct these employees to wear masks and either relocate them to a separate area or ensure they maintain a safe distance from others within the premises. Additionally, the company should assist these employees in seeking medical attention promptly or advise them to return home.
- Sick employees should wear masks and use tissue to cover their nose and mouth when coughing or sneezing. Used tissue should be promptly disposed of in a hands-free trash receptacle. If tissue is unavailable, they can use their elbows or shoulders to cover their mouth and nose, and they should prioritize hand hygiene.

1. **Educate employees on cough etiquette and hand hygiene**

- Display posters promoting compliance with cough etiquette and hand hygiene at prominent locations such as company entrances to encourage sick employees to stay home.
- Provide soap, water, or alcohol-based hand sanitizers in the workplace and ensure an ample supply. Hand sanitizers can be strategically placed in various locations or conference rooms to encourage employees to maintain hand hygiene.
- Instruct employees to wash their hands thoroughly with soap and running water for at least 20 seconds. Alternatively, they can use alcohol-based hand sanitizers containing at least 70% v/v ethanol to disinfect their hands. It’s essential to prioritize handwashing with soap and water if hands are visibly dirty.

1. **Regular environment clean and indoor air circulation maintain**

- Regularly clean all frequently touched surfaces in the workplace, including desktops, electronic devices, door handles, machine/electrical buttons, and switches, using cleaning agents commonly used in these areas. Follow label instructions for proper use.
- Unless specified by the Central Epidemic Command Center for high-risk areas, routine cleaning beyond disinfection measures is not necessary at this stage.
- Provide disposable tissues for employees to wipe surfaces before each use of frequently touched items, such as doorknobs, keyboards, remote controls, and desks.
- Maintain indoor air circulation by opening windows or vents to facilitate airflow. If using air conditioning, ensure at least one window is open to allow fresh air intake, leaving at least a fist-wide gap.
- Central air conditioning systems should increase the proportion of outdoor fresh air, minimize indoor air recirculation, and regularly replace or clean filters to ensure air quality.
  - 1. **Flexible measures for employees work and travel**

1. Refer to the CDC International Travel Recommendation Level Table to stay informed about the latest guidance and recommendations for the country of travel.
2. Prioritize the health and safety of workers amid the COVID-19 epidemic. If not essential, avoid sending workers to epidemic areas such as China. Utilize alternative methods such as video conferencing or telecommuting to maintain operations. Negotiate with workers to adjust work location and responsibilities as needed.
3. Ensure that employees understand the importance of promptly notifying their supervisor if they become ill while on business trips or temporary assignments. Inform them that they can call the epidemic prevention hotline 1922 for advice if necessary.
4. If employees fall ill while overseas, they should adhere to the company’s medical assistance policy. Alternatively, they can contact a local medical care provider, an overseas medical assistance company, or officials from their country’s embassy or consulate abroad for assistance in locating suitable medical care.
   - 1. **Measures for business continuity operations**
5. Develop a sustainable operation plan for the enterprise, appoint a responsible person for plan implementation, and provide relevant education and training to employees. Refer to the "Small and Medium Enterprises Continuous Operation Training Manual" for guidance.
6. Establish alternative mechanisms for decision-making authority and identify key technologies and personnel necessary for business continuity.
7. Formulate plans for off-site (remote) offices, off-site backup systems, alternative supply chains, and decentralized sources of raw materials and parts.
8. Develop solutions to meet the needs of important customers to ensure continued business relationships.
9. Utilize digital tools effectively to maintain trusted relationships with customers through video conferencing and increase online order intake through digital platforms.
10. For production and operations, procure machinery or information equipment to support remote work or telecommuting. Ensure access to material sources and logistics channels to facilitate transportation or emergency orders. Industries affected by the epidemic may partially suspend services while planning employee training, on-the-job training, or improvements to operational sites to enable swift resumption of operations post-epidemic and promote industry upgrades.
11. Take advantage of relevant government relief measures or resources to sustain basic operations, enhance operations, and improve competitiveness.
    - 1. **Other supporting policy measures**
12. For employees deemed at risk of infection based on epidemic investigation and risk assessment by the health unit, employers must ensure their absence from the workplace during the 14-day home quarantine period. However, flexible working arrangements such as telecommuting or conference calls can be adopted to allow employees to work from home.
13. If an employee is confirmed to be infected with SARS-CoV-2, the employer should cooperate with the health unit’s epidemic investigation and assess the risk of exposure for other colleagues in the workplace. Personal privacy must be protected in accordance with regulations. Based on risk assessment results, home isolation or independent health management will be implemented as per regulations.
14. Employees should be informed of cooperation requirements for home isolation, home quarantine, or independent health management through the latest information from the Central Epidemic Command Center, accessible at any time via their website (<https://www.cdc.gov.tw/>).
15. Workers must comply with health authorities’ directives for isolation or quarantine and are prohibited from working outside. Employers should grant quarantine leave for epidemic prevention, without penalizing workers with absenteeism, forced personal leave, or overtime. Employers may also apply for epidemic prevention compensation from the government during isolation or quarantine periods.
16. If a worker is determined to be infected with SARS-CoV-2 due to occupational reasons, the employer must grant public sick leave and provide wage compensation equivalent to the worker’s original salary. Additionally, if an employee suffers injury, disability, or death as a result, the employer must provide occupational accident compensation in accordance with the Labor Standards Act.
17. As of March 1, 2020, workers in medical institutions at risk of infection must wear masks at all times. In other industrial establishments with no infection risk, mask-wearing is not mandatory. However, employers cannot prohibit workers from wearing masks if they choose to do so. Public institution employers should align with information from the Central Epidemic Command Center and refer to guidelines from the Occupational Safety and Health Administration for workplace safety measures in response to severe infectious pneumonia. Timely revisions and adjustments should be made based on the epidemic’s severity to ensure worker safety and health.

*** Due to our study’s focus on the aforementioned content, we did not translate the remaining sections of this guideline.

# **Guideline 2**

**Guidelines on Occupational Safety and Health for Work from Home**

**Version: 2021.06.23**

1. **Introduction**

Amidst the COVID-19 pandemic and periods of community spread, the Central Epidemic Command Center has issued the “Guidelines for Enterprise Planning of Business Continuity in Response to COVID-19.” These guidelines advocate for remote work arrangements, adjusted attendance policies, and modified travel arrangements to mitigate the spread of the virus. Telecommuting, defined by the International Labour Organization (ILO) as a work arrangement where employees utilize information and communication technology (ICT) to perform assigned tasks from their homes, has emerged as a vital component of workplace epidemic prevention and response management.

However, it is crucial to recognize that the telecommuting environment and conditions often differ from traditional office settings. The purpose of this guideline is to provide considerations for occupational health and safety when implementing telecommuting measures. Enterprises are encouraged to identify potential hazards associated with telecommuting environments or tasks, conduct thorough risk assessments, and implement appropriate control measures based on the assessment results. Moreover, attention should be given to the physical and mental well-being of telecommuters, with periodic assessments and adjustments made to ensure optimal arrangements.

1. **Hazard identification and risk assessment for telecommuting**

Employers should collaborate with telecommuters to identify and assess potential occupational health and safety hazards, as well as the impact on physical and mental health, when performing their duties in their work environment. The following are examples:

1. Assess whether the nature of the work performed by the telecommuter is suitable for remote work. If not suitable, consider alternatives such as remote office relocation or staggered attendance.
2. Evaluate the health status of telecommuters to ensure they can work effectively in a suitable environment with adequate equipment support to maintain work safety and health.
3. Assess the surroundings, space, lighting, ventilation, flooring, electrical equipment, and facilities in the telecommuter’s work area for adequacy or potential hazards.
4. Evaluate the telecommuting mode and working conditions, including long working hours, abnormal workloads, or factors that may affect physical and mental health due to social isolation and an imbalance between work and life.
5. Identify any other factors that may jeopardize the personal safety and health of telecommuters.
6. **Safety and health management precautions**

Employers should, based on the characteristics of telecommuting work and the results of hazard risk assessments, implement necessary preventive measures or provide essential equipment or measures within a reasonable and feasible range. The attached checklist for safety and health management considerations includes the following key points:

**3.1. Necessary equipment, measures, or resources**

1. Employers should prioritize the elimination, replacement, engineering controls, administrative controls, and personal protective equipment for workplace hazards posed by telecommuting workers. This should be done considering existing technological capabilities and available resources. Employers should adopt control measures that can effectively reduce risks within a reasonable and feasible range.
2. Employers should provide telecommuters with necessary equipment and facilities or allow them to temporarily bring equipment from the workplace to the telecommuting location. This may include computers, monitors, keyboards, mice, printers, etc.
3. Telecommuting environments may not offer the same comprehensive working environment and facilities as regular workplaces. If there are concerns about the operation of information and communication tools or safety and health, employers should provide necessary resources and assistance.

**3.2. Home work area**

1. Encourage telecommuters to assess and maintain suitable telecommuting environments. Employers may offer appropriate guidance or assistance in this regard.
2. Ensure the safety, health, and comfort of the telecommuting workspace environment, including considerations for space, temperature, lighting, and ventilation as well as maintain cleanliness and tidiness of the work environment.
3. Prevent falls or slips by avoiding clutter or wetness in the flooring, aisles, or stairs of the workspace.
4. Minimize noise interruptions or distractions that may adversely affect telecommuters’ health during work.

**3.3. Work-related facilities**

1. Ensure that desks, chairs, monitors, keyboards, mice, etc., used for telecommuting are of appropriate types and heights, and the setup should be as ergonomic as possible.
2. Configure facilities to provide adequate stretching space, and encourage maintaining good posture during work to prevent musculoskeletal hazards
3. Provide support for using computer video equipment and software to ensure the stable operation of communication facilities such as the internet and telephone.
4. Ensure that electrical outlets and equipment are used normally and without damage or wear to prevent hazards such as electric shock or power overload.

**3.4. Physical and mental health management**

1. Telecommuting should avoid prolonged hours or excessive workloads. It is recommended to schedule regular rest breaks, such as stretching every half an hour to stand up and move around
2. Since telecommuting is typically solitary, it is important to implement relevant measures or engage in appropriate activities to mitigate psychological health risks such as isolation, depression, anxiety, and increased stress due to reduced interaction with colleagues or clients.
3. Telecommuters should prioritize their physical and mental health by maintaining regular exercise, adopting healthy eating habits, and ensuring adequate sleep.
4. It’s essential for telecommuters to maintain a balance between work and personal life. Establishing appropriate boundaries with partners, children, or roommates is recommended.

**3.5. Education and training**

1. Employers should offer necessary education and training to workers based on the characteristics of telecommuting work and the results of hazard risk assessments. This aims to enhance their awareness of hazards and equip them with basic knowledge of handling safety and health issues.
2. Guide telecommuters on conducting hazard risk assessments and establishing appropriate working equipment and facilities.
3. Provide information on common types of occupational musculoskeletal injuries during telecommuting and preventive measures.
4. Establish procedures for reporting accidents or suspected musculoskeletal injuries during telecommuting and contingency handling.
5. Offer guidance to telecommuters on planning appropriate work-rest schedules and maintaining good physical and mental health.
6. Provide education, training, and consultation assistance on the use of relevant information transmission equipment or tools for telecommuting.

**3.6. Communication and management**

1. Ensure that telecommuters can maintain effective communication with supervisors and colleagues through regular or ad-hoc video conferences, phone calls, or emails.
2. Support and promote informal communication and socialization among colleagues. Consider arranging regular online activities or facilitating interaction through social media platforms.

Telecommuters may have additional responsibilities, such as caring for family members who are also telecommuting, children engaged in distance learning, infants, elderly relatives, or family members with chronic illnesses. Employers should be flexible in adjusting working hours and work progress to accommodate such situations. Telecommuters should be informed of the employer’s expectations regarding work output and the necessity for adjustments and adaptations to temporary telecommuting arrangements.

1. Establish appropriate tracking management and safety and health audit mechanisms for telecommuting. Consider telecommuters’ feedback, conduct timely assessments, and implement rolling reviews and revisions as needed.
2. **Attachment**

Checklist for Safety and Health Management Precautions for Work from Home

**Attachment**

**Checklist for Safety and Health Management Precautions for Work from Home**

| Item | Checklist Content | |
| --- | --- | --- |
| 1. Necessary equipment, measures, or resources | - 1. □ Yes □ No | Have potential hazards associated with working from home been thoroughly assessed, and have appropriate equipment or measures been implemented to effectively mitigate these risks? |
|  | - 1. □ Yes □ No | Are essential equipment and facilities provided for home working, or are home workers permitted to temporarily relocate equipment from the workplace to their home working setup, including computers, monitors, keyboards, mice, printers, etc.? |
|  | - 1. □ Yes □ No | Are necessary resources and assistance available to home workers who have concerns regarding the operation of communication tools or their safety and health? |
| 1. Home work area | - 1. □ Yes □ No | Is there sufficient space designated for a home work area, allowing for adequate room to stretch comfortably? |
|  | - 1. □ Yes □ No | Are the temperature, lighting, and ventilation conditions in the home workspace satisfactory? |
|  | - 1. □ Yes □ No | Is the work area regularly maintained in a clean and organized manner, free from obstacles or slippery surfaces on floors and stairs? |
|  | - 1. □ Yes □ No | Is there any noise present in the work area that may disrupt work or impact physical and mental health? |
| 1. Work-related facilities | - 1. □ Yes □ No | Are the tables and chairs used by home workers set at appropriate heights, and do the chairs provide adequate support with armrests and backrests? |
|  | - 1. □ Yes □ No | Are the monitor height and the positioning of the keyboard, mouse, etc., adjusted appropriately for ergonomic comfort? |
|  | - 1. □ Yes □ No | Are the facilities required for working from home adequately spaced out and readily accessible? |
|  | - 1. □ Yes □ No | Are computer video equipment, software, and other necessary tools provided for telecommuting, along with resources to ensure stable operation of internet, telephone, and other communication facilities? |
|  | - 1. □ Yes □ No | Have electrical sockets and equipment been checked for normal use and any signs of damage or wear to prevent hazards such as electric shock or power overload? |
| 1. Physical and mental health management | - 1. □ Yes □ No | Assess whether home workers are experiencing continuous long working hours or abnormal workloads, and make necessary adjustments as needed. |
|  | - 1. □ Yes □ No | Confirm that home workers have appropriately planned their work and rest periods, such as incorporating regular breaks or engaging in stretching exercises every half hour. |
|  | - 1. □ Yes □ No | Ensure that relevant stress relief measures or suitable physical and mental health promotion activities are arranged for home workers. |
|  | - 1. □ Yes □ No | Check if home workers maintain regular exercise routines, adhere to healthy eating habits, and maintain a consistent sleep schedule. |
|  | - 1. □ Yes □ No | Assess if home workers are experiencing any physical symptoms that may require attention or intervention. |
| 1. Education and training | - 1. □ Yes □ No | Have home workers received necessary education and training tailored to the characteristics of their work and the results of hazard risk assessments? |
|  | - 1. □ Yes □ No | Will home workers receive necessary consultation and assistance to conduct hazard risk assessments? |
|  | - 1. □ Yes □ No | Are hazard prevention guidance and consultation provided for the more common types of occupational musculoskeletal injuries that may occur when working from home? |
|  | - 1. □ Yes □ No | Have home workers been educated, trained, or informed about reporting procedures and emergency response protocols in the event of an accident or suspected musculoskeletal injury while working from home? |
|  | - 1. □ Yes □ No | Is there provision for necessary education, training, or information dissemination to assist home workers in planning appropriate work and rest hours and maintaining good physical and mental health? |
|  | - 1. □ Yes □ No | Have home workers been provided with education, training, or consultation assistance on how to operate related information transmission equipment or software tools provided for telecommuting? |
| 1. Communication and management | - 1. □ Yes □ No | Have strategies been devised for supervisors or colleagues to maintain appropriate contact and communication with home workers? |
|  | - 1. □ Yes □ No | Are regular online communication or networking activities organized or supported for colleagues? |
|  | - 1. □ Yes □ No | Is necessary flexibility provided in working hours and progress to accommodate the practical needs of home workers’ spouses and family members (such as children attending remote classes, infants and young children needing care, elderly or chronically ill family members, etc.)? |
|  | - 1. □ Yes □ No | Has an appropriate tracking management and auditing mechanism been established for safety and health management of telecommuting, and is it regularly adjusted based on feedback from home workers? |
