# Supplementary material for: Effect of Government Guidelines and Corporate Governance on Telework Adoption and Occupational Health Measures in Taiwanese-Listed Companies
Source: Saf Health Work. 2024 May 7;15(2):164–71. doi: 10.1016/j.shaw.2024.04.004 (PMC11255928; doi:10.1016/j.shaw.2024.04.004)
Supplement: Multimedia component 2 [file mmc2.docx]

**Appendix 2**

Table. Cohen’s kappa values for internal consistency between observer and software that are rating the same items

| Questions and items | Human observer *vs*. Software | Human observer *vs*. Final result | Software *vs*. Final result |
| --- | --- | --- | --- |
| Adopting new working modes |  |  |  |
| Telework | 0.65 | 0.95 | 0.70 |
| Split operation | 0.69 | 0.92 | 0.77 |
| Work off-site | 0.63 | 0.83 | 0.78 |
| Flexible work time | 0.53 | 0.59 | 0.94 |
|  |  |  |  |
| Mentioning government guidelines | 0.71 | 1.00 | 0.71 |
|  |  |  |  |
| Providing telework support |  |  |  |
| Software and information security | 0.75 | 1.00 | 0.75 |
| Computer equipment | 0.82 | 1.00 | 0.82 |
| Online video training | 0.98 | 1.00 | 0.98 |
| Telework manual | 0.68 | 1.00 | 0.68 |
| Online consultation | 1.00 | 1.00 | 1.00 |
|  |  |  |  |
| Enhancing online communication |  |  |  |
| Videoconferencing software | 0.71 | 0.98 | 0.72 |
| Line | 0.84 | 0.85 | 0.99 |
| Voice dialing | 0.74 | 1.00 | 0.74 |
| E-mail | 0.86 | 1.00 | 0.86 |
|  |  |  |  |
| Assessing home-based telework environment | 1.00 | 1.00 | 1.00 |
|  |  |  |  |
| Implementing occupational health measures |  |  |  |
| On-site occupational health services | 0.68 | 1.00 | 0.68 |
| Online occupational health services | 0.73 | 0.87 | 0.87 |
| On-site mental health promotion activities | 0.68 | 0.83 | 0.85 |
| Online mental health promotion activities | 0.54 | 0.89 | 0.72 |
| Fitness facilities and activities | 0.69 | 1.00 | 0.69 |
| Training on taking rest breaks at work | 1.00 | 1.00 | 1.00 |
